# Experimental Investigation of One-Way Lamb and SH Mixing Method in Composite Laminates

**DOI:** 10.3390/s25247631

**Published:** 2025-12-16

**Authors:** Siyang Xie, Youxuan Zhao, Yuzi Liu

**Affiliations:** 1 College of Aerospace Engineering, Chongqing University, Chongqing 400044, China; 202431131055@stu.cqu.edu.cn; 2 China Aircraft Intensity Research Institute, Xi’an 710065, China; liuyuzi15735185412@163.com

**Keywords:** ultrasonic nonlinearity, Lamb wave, wave mixing, composite laminates, damage localization

## Abstract

This paper experimentally investigates the resonant behavior of the one-way Lamb and SH (shear horizontal) mixing method in composite laminates with inherent quadratic nonlinearity, delamination damage and impact damage. When the fundamental *S*_0_-mode Lamb waves and *SH*_0_ waves mix in the damage regions of composite laminates, experimental results demonstrate the generation of the resonant *SH*_0_ waves with the resonance condition. Meanwhile, the damage localization method in composite laminates is experimentally verified by the time-domain signal of resonant waves. Furthermore, it is found that the one-way Lamb and SH mixing method is sensitive to inherent quadratic nonlinearity and impact damage but insensitive to delamination damage.

## 1. Introduction

The application of composite laminates has grown significantly in aeronautical and other industries due to their excellent mechanical properties, lightweight characteristics and corrosion resistance. However, different types of damage could gradually lead to the performance degradation of composite laminates in service, which are mainly fiber fracture, matrix cracking, matrix–fiber interface debonding and delamination [1]. The development of performance degradation could result in a final failure. Therefore, it is essential to detect and evaluate performance degradation for ensuring the integrity and durability of composite structures.

Non-destructive testing (NDT) technology is widely used in composite material testing applications [2,3], including the ultrasonic method [4,5,6,7,8], X-ray detection method [9,10], infrared thermal imaging method [11,12] and acoustic emission (AE) method [13,14]. Among them, the ultrasonic method has attracted wide attention due to its high sensitivity and strong penetration even in composite structures, which includes the linear ultrasonic method [15,16] and nonlinear ultrasonic method [17,18,19]. Furthermore, nonlinear guided wave techniques have great potential to evaluate early material degradation in composite laminates with their long propagation and high sensitivity to material microstructures. Considering different nonlinear effects, the higher harmonics technique [20,21], nonlinear wave mixing technique [22,23,24] and quasi-static component (QSC) technique [25,26] have been widely studied in recent years.

Many efforts have been devoted to the application of the higher harmonics technique in composites. Shan et al. [27] have proposed two damage indices to evaluate delamination in composites based on the amplitude of second harmonic Lamb waves. Rauter et al. [28] have applied a second harmonic guided wave to detect fatigue damage in composites. Alnuaimi et al. [29] have proposed the modified Sideband Peak Count Index (SPC-I) technique to detect and monitor impact damage in composite plates. During the propagation of nonlinear ultrasonic waves, the quasi-static component can also be generated with the generation of higher harmonics. Wang et al. [30] have employed QSC to evaluate the thickness of materials with high attenuation. Li et al. [25] have experimentally evaluated thermal degradation by detecting QSC pulses. Jiang et al. [31] have investigated static component (SC) generation of guided waves (GWs) under group velocity matching condition to assess hygrothermal damage and low-velocity impact damage in composite plates.

Although the higher harmonics technique and quasi-static component technique are able to evaluate nonlinear damage, the nonlinear wave mixing technique has received more attention for its abilities of locating damage positions and avoiding interference from noise signals. The nonlinear wave mixing method with bulk wave has been employed to detect plasticity, fatigue, corrosion [32] and thermal aging [33] in materials. Sun et al. [34] have analytically derived and numerically investigated the resonance conditions for one-way resonant mixing of nonlinear Lamb waves. Lissenden et al. [35] have proposed internal resonance criteria for the guided wave-mixing method: phase–velocity matching and the nonzero power flux. Li et al. [36] have proposed the one-way *S*_0_-*A*_0_ mixing method with the resonance condition $\omega_{S_{0}}/\omega_{A_{0}}=2\kappa /\left(\kappa +1\right)$ to maximize the energy of the resonant wave (where $\kappa =c_{S_{0}}/c_{A_{0}}$, $c_{S_{0}}$ and $c_{A_{0}}$ are the phase velocities of the *S*_0_ and *A*_0_ mode Lamb waves) and experimentally investigated the corrosion damage evaluation [37]. Jiao et al. [38] have experimentally and numerically investigated residual stress measurement in metal plates based on the collinear Lamb wave mixing method. Li et al. [39] have experimentally investigated zero-group velocity (ZGV)-combined harmonic generation induced by the mixing of counter-directional Lamb waves. Zhu et al. [40] have explored the low-frequency Lamb wave mixing method for fatigue damage evaluation. Liu et al. [41] have proposed and verified the one-way Lamb and SH mixing method for evaluating and locating the damage region in thin plates. Shan et al. [42] have investigated the generation of cumulative secondary *S*_0_ Lamb waves at the sum frequency when two primary codirectional shear-horizontal *SH*_0_ waves converge. Based on the progress in guided wave mixing technique research, recent work has begun exploring its applicability to composite materials. Li et al. [43] have employed the guided wave mixing technique combined with the mixing frequency peak counting approach to evaluate impact damage in composites. Pineda et al. [44] have applied the *A*_0_-*A*_0_ Lamb mixing method to detect delamination damage in composite laminates. Lan et al. [45] have employed the chirp guided wave mixing method (CGWM) to detect delamination in composite laminates. Aslam et al. [46] have investigated the nonlinear acoustic response of feature-guided waves (FGWs) in welded joints by one-way and two-way mixing methods.

Compared with other guided wave mixing methods, the *S*_0_-*SH*_0_ mixing method could have great potential for damage evaluation due to the approximate non-dispersion characteristic of *SH*_0_ waves in the low-frequency range [47], which could reduce the complexity of ultrasonic signal processing. Recently, the *S*_0_-*SH*_0_ mixing method has been numerically and experimentally investigated on a 2 mm aluminum plate [41]. Considering the anisotropy of composite laminates, the feasibility of the *S*_0_-*SH*_0_ mixing method needs to be discovered through numerical simulations [48] and experimental measurements. Therefore, this paper aims to experimentally investigate the feasibility of the one-way Lamb and SH mixing method for the damage detection of composite laminates with inherent quadratic nonlinearity, delamination damage and impact damage. Meanwhile, the method to locate the impact damage region in composite laminates is investigated, and the result is compared with a C-scan result. The novelty of this paper could be the experimental demonstration of the feasibility of the one-way *S*_0_-*SH*_0_ mixing method to evaluate damage in composite laminates.

## 2. Lamb and SH Mixing Method in Composite Laminates

A transverse wave pulse and a longitudinal wave pulse are successively emitted at the same side and propagate in the same direction with the resonance condition $\omega_{L}/\omega_{T}=2\kappa /\left(\kappa +1\right)$; this process is called the one-way mixing technique [49], where $\kappa =c_{L}/c_{T}$, $c_{L}$ and $c_{T}$ are the longitudinal and transverse velocities, and $\omega_{L}$ and $\omega_{T}$ are the circular frequencies of the longitudinal and transverse wave pulses, respectively. Then, when two fundamental wave pulses mix in the region with quadratic nonlinearity, a transverse resonant wave with difference frequency of these wave pulses can be generated and propagate in the opposite direction of the fundamental transverse wave pulse, where quadratic nonlinearity derives from the quadratic nonlinear elastic constitutive model, including second-order and third-order constants [41]. Similarly, the one-way *S*_0_-*A*_0_ mixing method with the resonance condition $\omega_{S_{0}}/\omega_{A_{0}}=2\kappa_{1}/\left(\kappa_{1}+1\right)$ and the one-way *S*_0_-*SH*_0_ mixing method with the resonance condition $\omega_{S_{0}}/\omega_{SH_{0}}=2\kappa_{2}/\left(\kappa_{2}+1\right)$ are successively proposed and verified by Zhao et al. [37,41], where $\kappa_{1}=c_{S_{0}}/c_{A_{0}}$ and $\kappa_{2}=c_{S_{0}}/c_{SH_{0}}$, $c_{S_{0}}$, $c_{A_{0}}$ and $c_{SH_{0}}$ are the phase velocities of the *S*_0_- and *A*_0_-mode Lamb waves and *SH*_0_ waves, respectively.

**Figure 1:**
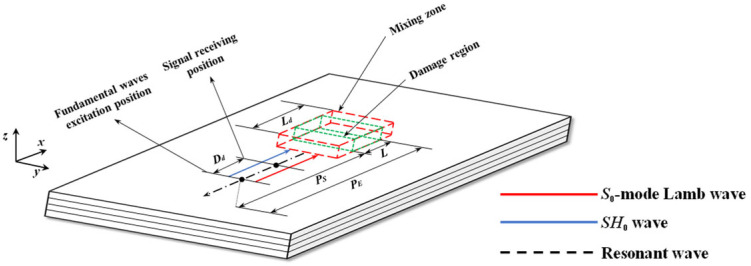
Schematic of one-way mixing of *S*_0_-mode Lamb waves and *SH*_0_ waves in composite laminates with quadratic nonlinearity. Red box—mixing zone, green box—damage region.

Considering composite laminates with quadratic nonlinearity, the one-way Lamb and SH mixing method is also effective. The schematic of the one-way *S*_0_-*SH*_0_ mixing method in composite laminates with quadratic nonlinearity is described in Figure 1. To locate the damage region with the length *L*, *SH*_0_ wave pulses and *S*_0_-mode Lamb wave pulses are sequentially excited in a fundamental waves excitation position. Then, they interact with each other in the mixing zone with the length *L_d_*. When the resonance condition $\omega_{S_{0}}/\omega_{SH_{0}}=2\kappa_{2}/\left(\kappa_{2}+1\right)$ is satisfied and the quadratic nonlinearity of composite laminates exists, a series of nonlinear effects could be triggered, which leads to the generation of the resonant *SH*_0_ wave. The frequency of the resonant *SH*_0_ wave is $\omega_{R}=\omega_{S_{0}}-\omega_{SH_{0}}$, and its propagation direction is opposite to that of fundamental *SH*_0_ waves, where $\omega_{S_{0}}$ and $\omega_{SH_{0}}$ are the circular frequencies of *S*_0_-mode Lamb waves and *SH*_0_ waves, respectively. The resonant *SH*_0_ wave is finally received at the signal-receiving position, which is *D_d_* away from the fundamental waves excitation position.

Then, the damage localization formula based on the heterodyne detection technique can be expressed by Equations (1)–(3) [41]:

(1) $$P_{S}=(T_{start}-\Delta T+D_{d}/V_{g}^{SH_{R}})V_{g}^{S}V_{g}^{SH_{R}}/\left(V_{g}^{S}+V_{g}^{SH_{R}}\right)$$

(2) $$P_{E}=(T_{end}-T_{S}-\Delta T+D_{d}/V_{g}^{SH_{R}})V_{g}^{S}V_{g}^{SH_{R}}/\left(V_{g}^{S}+V_{g}^{SH_{R}}\right)$$

(3) $$L=P_{E}-P_{S}$$

where $P_{S}$ is the distance between the left boundary of the damage region and fundamental waves excitation position in Figure 1, $P_{E}$ is the distance between the right boundary of the damage region and fundamental waves excitation position, *L* is the length of the damage region, *D_d_* is the distance between fundamental waves excitation position and receiving position, $T_{S}$ is the time of exciting fundamental *S*_0_-mode Lamb wave pulses, ΔT is the time interval between exciting *S*_0_-mode Lamb wave pulses and *SH*_0_ wave pulses, $V_{g}^{SH_{R}}$ is the group velocity of the resonant wave, $V_{g}^{S}$ is the group velocity of the fundamental *S*_0_-mode Lamb waves, and $T_{start}$ and $T_{end}$ are the start and the end time of the resonant wave, respectively.

## 3. Experimental Measurement

In this section, the feasibility of the one-way *S*_0_-*SH*_0_ mixing method to detect damage regions in composite laminates is explored through experimental measurements. Inherent quadratic nonlinearity, delamination damage and impact damage are considered, respectively. Meanwhile, the effectiveness of one-way *S*_0_-*SH*_0_ and *S*_0_-*A*_0_ mixing methods for delamination damage detection is also investigated. In addition, the reliability of the damage localization method for impact damage in composite laminates is verified.

### 3.1. Experimental Setup

#### 3.1.1. Preparation of Composite Samples with Damage

In this study, experimental measurements are conducted on T300 carbon-fiber-reinforced composite laminates (Blue Label Composite Materials Inc., Dezhou, China), which have dimensions of 1000 mm × 400 mm × 3.2 mm with the stacking sequence [0/90]_4s_.

Three cases are prepared to investigate the feasibility of the one-way *S*_0_-*SH*_0_ mixing method for detecting damage regions in three individual composite laminates.

**Figure 2:**
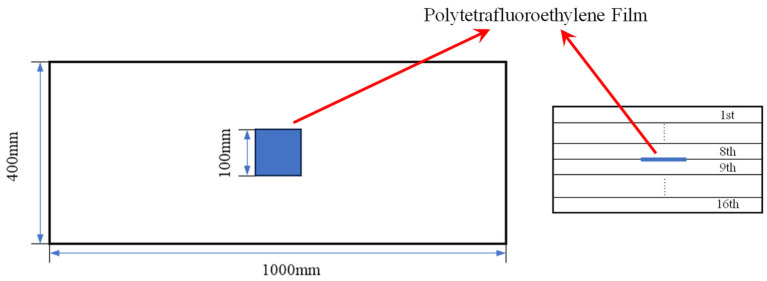
Schematic of delamination damage in composite laminates ((**Left**): layout diagram; (**Right**): the position of polytetrafluoroethylene film in the thickness direction).

1. For case A, inherent quadratic nonlinearity of composite laminates is introduced as one type of early material degradations, which is naturally caused by the manufacturing process [50]. Thus, case A, with inherent quadratic nonlinearity, is employed to verify the generation mechanism and the resonance condition of the one-way *S*_0_-*SH*_0_ mixing method in composite laminates.
2. For case B, delamination damage in composite laminates is introduced by prefabricated delamination, which is arranged at the center of the composite sample and between the 8th and 9th layers through a 100 × 100 mm polytetrafluoroethylene (also known as Teflon [51]) film, which is effective in separating two mating surfaces without significantly altering the properties of the composite laminates [52]. The schematic of delamination damage in composite laminates is shown in Figure 2. Meanwhile, the one-way *S*_0_-*SH*_0_ mixing method and the one-way *S*_0_-*A*_0_ mixing method are both employed to detect delamination damage, and the comparison of their effects are discussed in detail.
3. For case C, impact damage with an impact energy of 10 J [53,54] is introduced in composite laminates by dropping a 1 kg weight and 62.5 mm diameter iron ball from a 1 m height position, which is employed to verify the capability of the one-way *S*_0_-*SH*_0_ mixing method for detecting and locating impact damage.

#### 3.1.2. Ultrasonic Measurement

**Figure 3:**
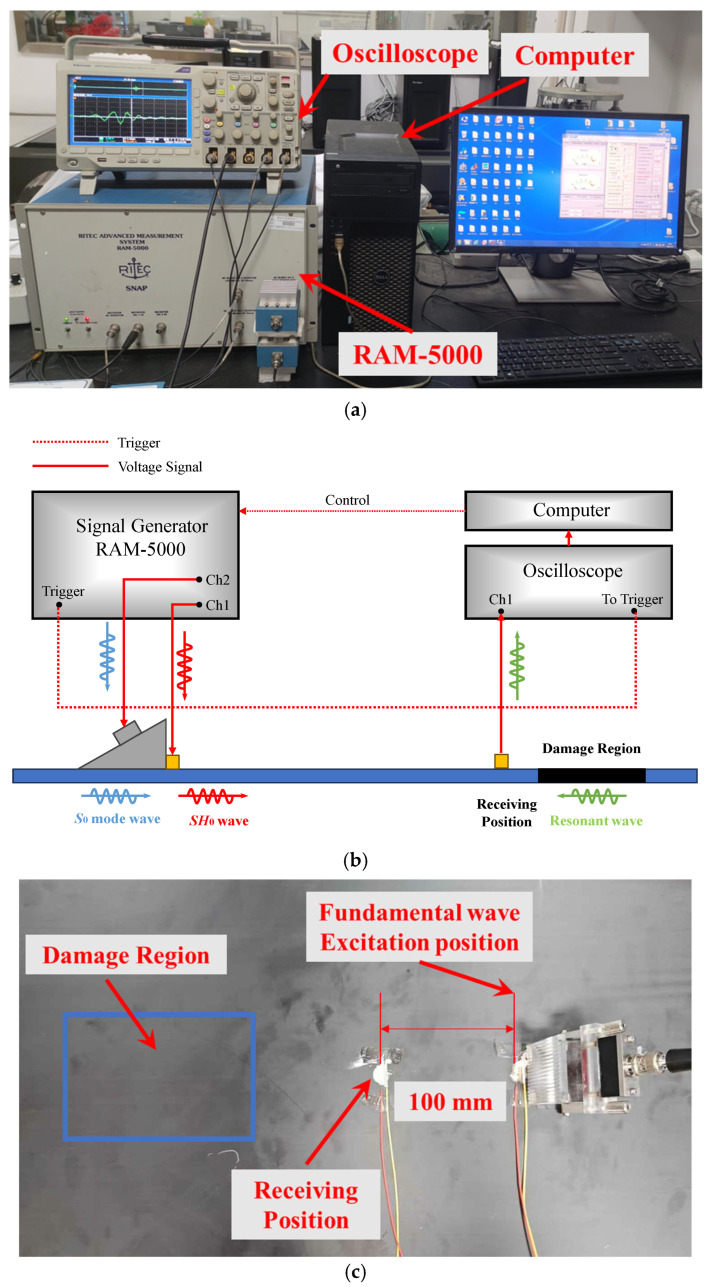
Ultrasonic experimental setup. (**a**) Nonlinear ultrasonic experimental system: RAM-5000, signal generator; Oscilloscope, signal receiver; Computer, signal processor; (**b**) schematic diagram of ultrasonic experimental setup, corresponding to the ultrasonic experimental system; (**c**) layout of composite laminate in ultrasonic experiments.

The experimental setup of the *S*_0_-*SH*_0_ mixing method in composite laminates is shown in Figure 3. The fundamental signals are generated by two channels, respectively, from the RAM-5000 SNAP system (RITEC Inc., Warwick, RI, USA). Channel 1 is applied to excite *SH*_0_ waves by the PZT-5H piezoelectric ceramic(d_15_) [55] (Baoding Hongsheng Ceramic Inc., Baoding, China). According to the theoretical dispersion curve, the excited frequencies of *SH*_0_ waves in the low-frequency range (100 kHz–250 kHz) are selectively employed to ensure only zero-mode *SH* waves in experiments. Then, channel 2 is applied to excite *S*_0_-mode Lamb waves by Olympus transducer (Model: A413S-SB, Olympus Inc., Tokyo, Japan) through the wedge block (Model: ABWX-2001, Olympus Inc., Tokyo, Japan) with an oblique angle of 26.2° [41]. The PZT-5H piezoelectric ceramic and the wedge block are located at excitation positions with zero distance. Meanwhile, the transducer, wedge, and composite laminates are coupled with an ultrasonic coupling agent. The PZT-5H piezoelectric ceramic is bonded to the surface of the composite laminate using conductive silver adhesive. When *SH*_0_ waves and *S*_0_-mode Lamb waves interact with each other in the damage region, the resonant waves can be generated and propagate in the opposite direction of two fundamental waves, which can be received and converted into a voltage signal by another PZT-5H piezoelectric ceramic at the receiving position. The distance between the wave excitation position and receiving position is fixed at 100 mm. Finally, to reduce uncertainty, the voltage signals are received by the DPO 3014 digital oscilloscope (Tektronix Inc., Beaverton, OR, USA) with 512 times sampling average operation, which is saved into the computer. Note that the size and location of the damage region in three cases are different. Therefore, the damage region in Figure 3 is shown for illustrative purposes only.

The frequencies of two fundamental waves are selected according to dispersion curves (discussed in Section 3.2.1 in detail), and both fundamental wave pulses have 10 cycles with a continuous sinusoidal signal [37]. In addition, the synthesizer amplitude for *SH*_0_ waves is set as 50%; the output level of gated amplitude is set as 50. For *S*_0_-mode Lamb waves, the synthesizer amplitude and the output level of gated amplitude are the same as for *SH*_0_ waves.

The signal processing method in Ding’s experiment [37] is employed to effectively obtain the resonant wave signals. The received signal *α* is acquired by only emitting *SH*_0_-mode fundamental waves, then the received signal *β* is acquired by only emitting *S*_0_-mode fundamental waves; finally, the received signal *γ* is acquired by emitting both *S*_0_- and *SH*_0_-mode fundamental waves. As a result, the resonant wave signal *η* = signal *γ* − signal *α* − signal *β*.

### 3.2. Experimental Results

#### 3.2.1. The Selection of Fundamental Waves

**Figure 4:**
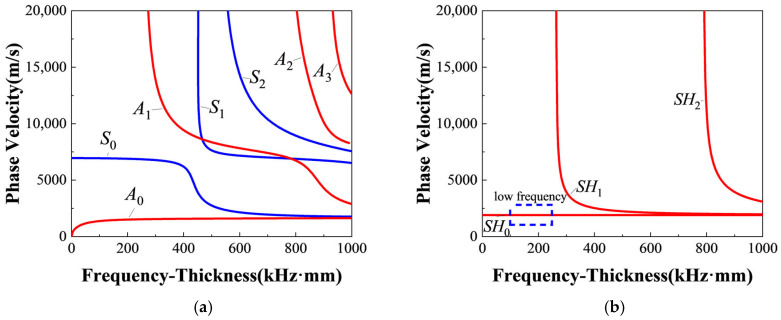
The dispersion curves of T300 composite laminates (only phase velocity): (**a**) Lamb waves; (**b**) SH waves.

The frequencies of *S*_0_-mode Lamb waves and *SH*_0_ waves are vital for the generation of resonant waves, which need to be carefully selected. The frequency pairs of two fundamental waves need to satisfy the resonance condition $\omega_{S_{0}}/\omega_{SH_{0}}=2\kappa /\left(\kappa +1\right)$ and only generate *S*_0_-mode Lamb waves and *SH*_0_ waves. Considering the material properties of T300 carbon-fiber-reinforced composite listed in Table 1 (from the software Dispersion Calculator V2.0 [56]), the dispersion curves of Lamb waves and SH waves in a 3.2 mm thick T300 composite laminates are shown in Figure 4. Therefore, the representative frequency pairs are carefully selected and listed in Table 2, where $f_{S_{0}}$ and $f_{SH_{0}}$ are, respectively, the frequencies of *S*_0_-mode Lamb waves and *SH*_0_ waves; $f_{R}=f_{S_{0}}-f_{SH_{0}}$ is the theoretical frequency of the resonant waves.

**Figure 5:**
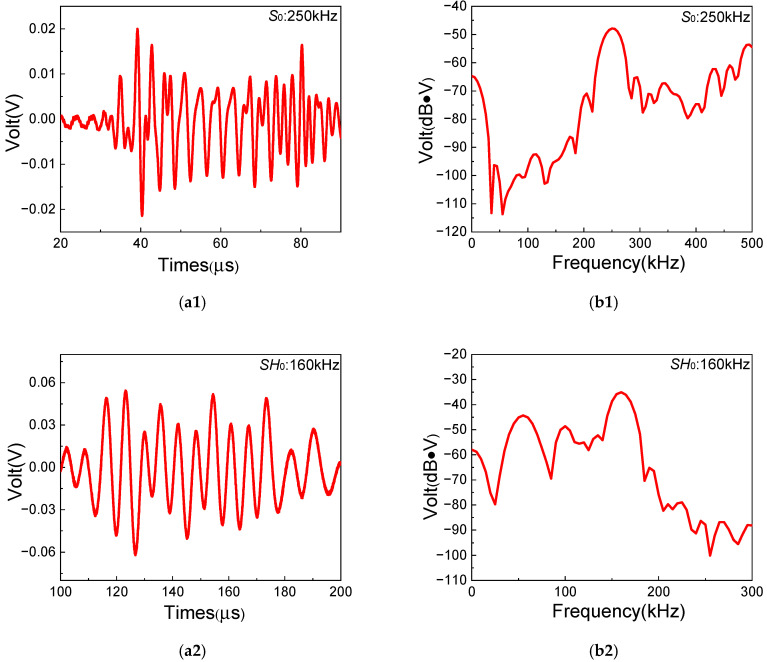
Time-domain and frequency-domain signals of two fundamental waves collected in the experiment: (**a1**,**a2**) 250 kHz *S*_0_-mode Lamb waves; (**b1**,**b2**) 160 kHz *SH*_0_ waves.

More importantly, the frequencies and group velocities of two fundamental waves need to be prudently obtained to verify the generation of *SH*_0_ waves and *S*_0_-mode Lamb waves in experiments. Figure 5 shows the typical time-domain and frequency-domain signals of fundamental *SH*_0_ waves and *S*_0_-mode Lamb waves ($f_{S_{0}}$ = 250 kHz and $f_{SH_{0}}$ = 160 kHz). The frequency-domain signals are obtained by performing FFT on time-domain signals. Then, it can be found that the experimental frequencies agree well with excited frequencies of fundamental waves. The experimental results of other frequency pairs listed in Table 2 are also as expected. Meanwhile, the group velocities of fundamental waves with different frequencies are listed in Table 3 and Table 4. The theoretical group velocities can be determined from dispersion curves, and the experimental group velocities can be calculated by Time-of-Fly of wave packets between two fixed points [57]. Then, the approximate non-dispersion characteristic of *SH*_0_ waves and low-level dispersion characteristic of *S*_0_-mode Lamb waves in low-frequency range can be verified in quasi-isotropic composite laminates. Moreover, it can be seen that the error of *SH*_0_ waves’ group velocities between experiment and theory decreases with the increase in the frequency, while the error of group velocity of *S*_0_-mode Lamb waves between experiment and theory changes within a small range. The errors could be caused by the deviation between actual material properties in experimental composite laminates and theoretical material properties listed in Table 1. The experimental group velocities are also in good agreement with the theoretical group velocities from the dispersion curves in Figure 4. Therefore, it can be confirmed that *S*_0_-mode Lamb waves and *SH*_0_ waves in composite laminates are successfully excited by the Olympus transducer and the PZT-5H piezoelectric ceramic, respectively.

#### 3.2.2. Results of Case A

**Figure 6:**
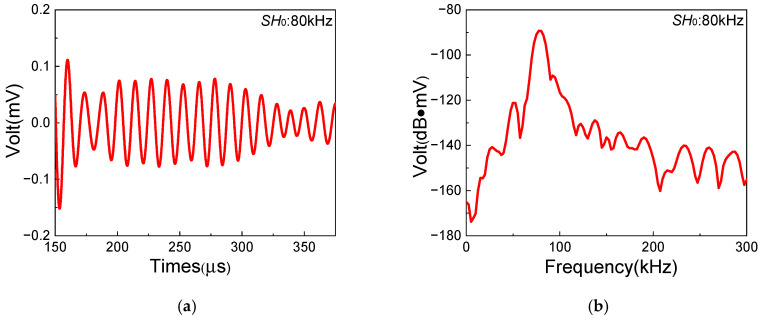
Resonant wave signals with the frequency 80 kHz, generated from frequency pair of 140 kHz *SH*_0_ fundamental wave and 220 kHz *S*_0_-mode fundamental wave: (**a**) time domain; (**b**) frequency domain.

**Figure 7:**
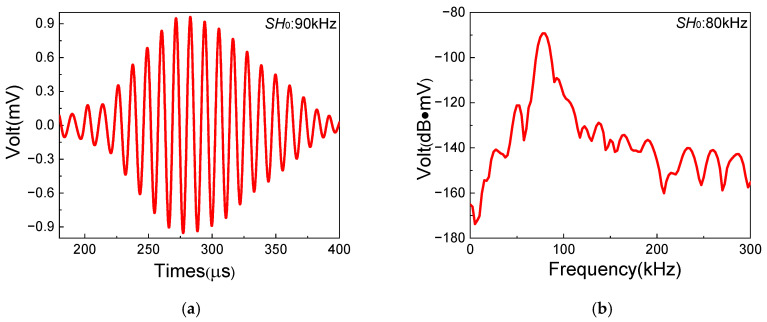
Resonant wave signals with the frequency 90 kHz, generated from frequency pair of 160 kHz *SH*_0_ fundamental wave and 250 kHz *S*_0_-mode fundamental wave: (**a**) time domain; (**b**) frequency domain.

**Figure 8:**
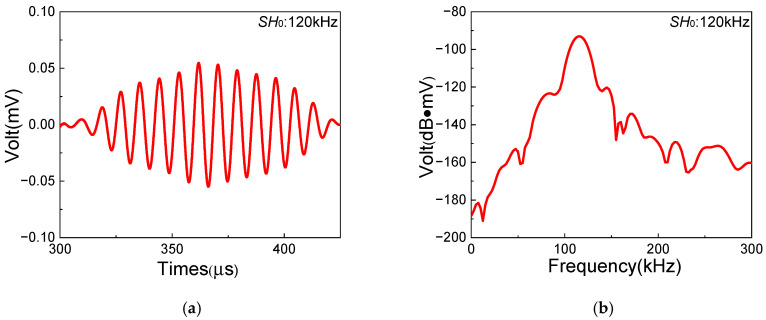
Resonant wave signals with the frequency 120 kHz, generated from frequency pair of 210 kHz *SH*_0_ fundamental wave and 330 kHz *S*_0_-mode fundamental wave: (**a**) time domain; (**b**) frequency domain.

In this section, a series of experiments based on the one-way *S*_0_-*SH*_0_ mixing method with different frequency pairs are conducted in composite laminates with inherent quadratic nonlinearity, which aim to investigate the feasibility of the generation of resonant waves. The frequencies and waveforms of resonant waves are obtained to verify the generation of *SH*_0_ resonant waves. Figure 6, Figure 7 and Figure 8 show the time-domain and frequency-domain signals of resonant waves with different frequencies. Note that, considering the frequency bandwidth from ($f_{R}$ − 10) kHz to ($f_{R}$ + 10) kHz, a Butterworth filter is applied to clearly obtain resonant wave signals. Distinct diamond shapes could be found in the waveforms of resonant waves, which is consistent with the results of numerical simulation from Liu [48]. Then, the frequencies of different resonant waves can be obtained, as listed in Table 5. It is found that the errors of resonant frequencies between experiment and theory change within a small range. Meanwhile, resonant waves collected at receiving position can also prove the opposite propagation direction of fundamental waves, which is located between the excitation position and the damage region. Therefore, it is experimentally confirmed that resonant waves can be successfully generated in composite laminates with inherent quadratic nonlinearity when the resonance condition $\omega_{S_{0}}/\omega_{SH_{0}}=2\kappa /\left(\kappa +1\right)$ is satisfied.

**Figure 9:**
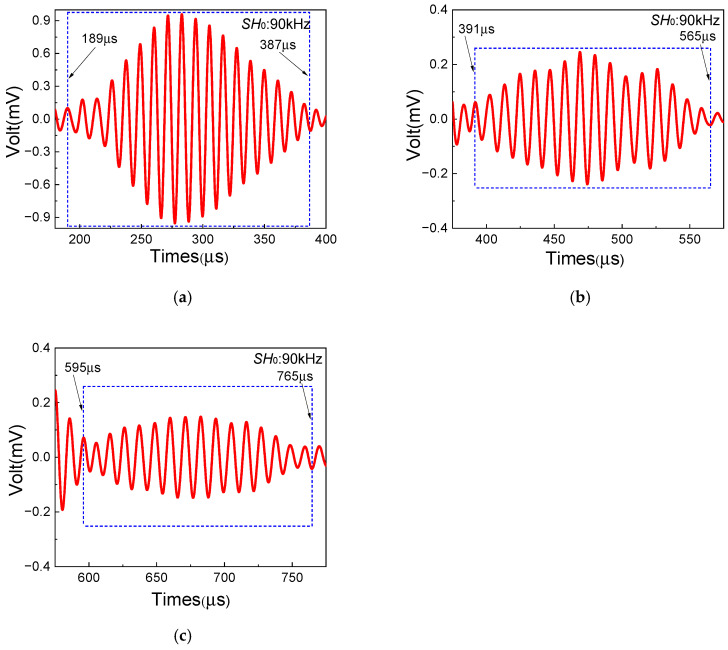
Resonant wave signals with the frequency 90 kHz with different delay times: (**a**) 120 μs, (**b**) 200 μs, (**c**) 260 μs. The left and right ends of the blue dashed box indicate the start and end times of resonant wave signals.

Additionally, to further validate the damage localization method in Section 2, the frequency pair of $f_{S_{0}}$ = 250 kHz and $f_{SH_{0}}$ = 160 kHz is adopted to investigate the start and end time of resonant waves. Based on the one-way *S*_0_-*SH*_0_ mixing method, the mixing zone of resonant waves can be controlled by changing the delay time. Thus, the intervals between *S*_0_-mode Lamb wave and *SH*_0_ wave excitation time are set to 120 μs, 200 μs and 260 μs, respectively. Figure 9 shows the time-domain signals of resonant waves with different intervals, clearly indicating the start and end time. Note that *T_start_* in experiments shows the start time of resonant waves when the variation magnitude of the amplitude of the time-domain signal suddenly increases, and *T_end_* shows the end time of resonant waves when the variation magnitude of the amplitude of the time-domain signal suddenly decreases. The localization results with the comparison between experiment and theory are listed in Table 6. Small errors between experimental values and theoretical values can be found. Then, the position and the length of the damage region in composite laminates can be accurately and experimentally obtained through time-domain signals of resonant waves. Therefore, based on the one-way *S*_0_-*SH*_0_ mixing method, the damage localization in composite laminates with inherent quadratic nonlinearity can be achieved with remarkable potential.

#### 3.2.3. Results of Case B

Different from case A, composite laminates for case B with delamination damage aim to investigate the feasibility of delamination damage detection in composite laminates based on the *S*_0_-*SH*_0_ mixing method. It should be noted that, due to appropriate sensitivity and intensity, the frequency pair ($f_{S_{0}}$ = 250 kHz and $f_{SH_{0}}$ = 160 kHz) is employed in the following experiments.

**Figure 10:**
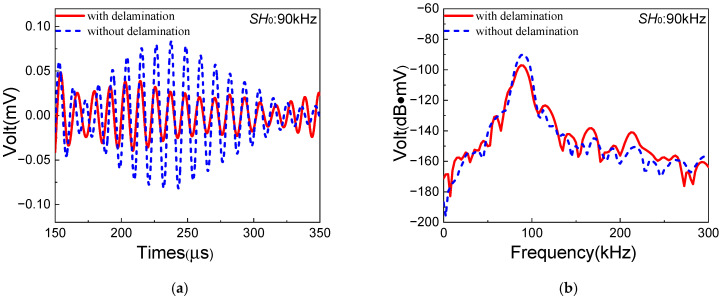
Resonant wave signals with the frequency 90 kHz generated from *S*_0_-*SH*_0_ mixing experiments in composite laminates with/without delamination: (**a**) time domain; (**b**) frequency domain.

The resonant wave signals in composite laminates with/without delamination are shown in Figure 10. Due to delamination damage, the amplitude of resonant waves decreases remarkably with distinct degradation of the diamond-shaped waveform. Meanwhile, the ANP (acoustic nonlinearity parameter) [41] is defined as $\beta =A_{R}/\left(A_{S_{0}}\cdot A_{SH_{0}}\right)$, where $A_{R}$, $A_{S_{0}}$ and $A_{SH_{0}}$ are the amplitudes of resonant waves, *S*_0_-mode Lamb waves and *SH*_0_ waves, respectively. In this section, the ANPs without delamination and with delamination are denoted as $\beta_{i}$ and $\beta_{d}$, respectively. Delamination damage in composite laminates is cumulatively generated on the basis of inherent quadratic nonlinearity. Thereby, the ANP focused on delamination damage is denoted as $\beta_{d}^{\prime }=\beta_{d}-\beta_{i}$. The experimental values of $\beta_{i}$ and $\beta_{d}^{\prime }$ are 3.24 and −0.68, respectively, which means that ANP observably decreases with delamination damage. It could be inferred that the one-way *S*_0_-*SH*_0_ mixing method is not sensitive to delamination damage in composite laminates. The vibration direction of *SH*_0_ waves is perpendicular to the direction of delamination type I, which could lead to ineffective energy transmission of fundamental *SH*_0_ waves to the delamination damage. Thus, lower-level resonant waves could be generated in the process, which could be the possible reason.

**Figure 11:**
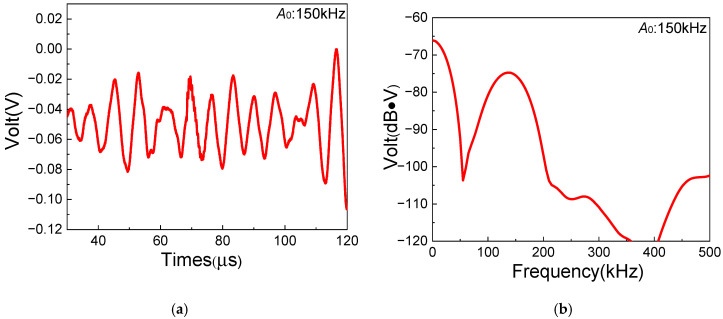
*A*_0_-mode fundamental wave signals with the frequency 150 kHz collected in the experiment: (**a**) time domain (with 1 MHz Butterworth filtering processing); (**b**) frequency domain.

To verify the above inference, a similar experiment based on the one-way *S*_0_-*A*_0_ mixing method is employed for comparison. Similarly to the experiments of the one-way *S*_0_-*SH*_0_ mixing method, the selection of fundamental frequency pairs of the one-way *S*_0_-*A*_0_ mixing method is also based on the dispersion curves and the resonance condition [36]. Thus, 250 kHz of the *S*_0_-mode Lamb wave and 150 kHz of the *A*_0_-mode Lamb wave are appropriately selected for the *S*_0_-*A*_0_ mixing experiment. As a result, a 100 kHz *A*_0_ resonant wave could be generated in composite laminates. Note that the *A*_0_-mode wave with the frequency of 150 kHz is excited via another Olympus transducer (Model: A413S-SB) at the excitation position [37]. Figure 11 shows the time-domain and frequency-domain signals of 150 kHz *A*_0_-mode fundamental Lamb waves with 1 MHz filtering processing, which could reduce the oscillations. The experimental frequencies agree well with excited frequencies of fundamental waves. Compared with the theoretical group velocity of *A*_0_-mode Lamb waves at 1572.63 m/s, the experimental value is 1654.56 m/s, which shows a small error of 5.21%. Therefore, it can be confirmed that *A*_0_-mode Lamb waves in composite laminates are successfully excited by the Olympus transducer.

**Figure 12:**
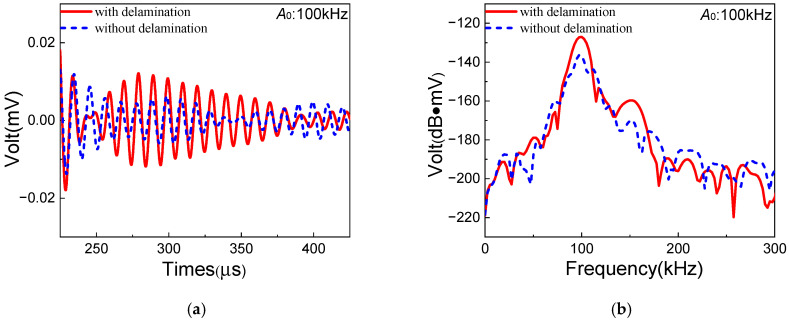
Resonant wave signals with the frequency 100 kHz generated from *S*_0_-*A*_0_ mixing experiments in composite laminates with/without delamination: (**a**) time domain; (**b**) frequency domain.

Next, the one-way *S*_0_-*A*_0_ mixing experiments are conducted in the composite laminates without/with delamination, respectively. The signals of corresponding resonant waves are shown in Figure 12. The amplitude of *A*_0_ resonant waves increases significantly with the distinct diamond-shaped waveform. Meanwhile, the ANP for the one-way *S*_0_-*A*_0_ mixing method is defined as $\beta =A_{R}/\left(A_{S_{0}}\cdot A_{A_{0}}\right)$ [36], where $A_{A_{0}}$ is the amplitude of *A*_0_-mode Lamb waves. And the similar ANPs $\beta_{i}$, $\beta_{d}$ and $\beta_{d}^{\prime }$ are also adopted in the *S*_0_-*A*_0_ mixing experiments. The comparison of ANPs between *S*_0_-*SH*_0_ and *S*_0_-*A*_0_ mixing methods is shown in Table 7. It can be found that the ANP $\beta_{d}^{\prime }$ of the *S*_0_-*A*_0_ mixing method increases significantly compared with that of the *S*_0_-*SH*_0_ mixing method. The vibration direction of the *A*_0_ resonant wave is parallel to the direction of delamination type I, which leads more sensitivity to the delamination damage area in composite laminates of the one-way *S*_0_-*A*_0_ mixing method. Thus, considering delamination damage in composite laminates, it is suggested that the *S*_0_-*A*_0_ mixing method could be more appropriate than the *S*_0_-*SH*_0_ mixing method.

#### 3.2.4. Results of Case C

Different from cases A and B, impact damage is introduced to investigate the feasibility of the *S*_0_-*SH*_0_ mixing method in detecting impact damage of composite laminates. Based on the research results from References [53,54], the energy limit of barely visible impact damage (BVID) is approximately 5–15 J, and the approximate circular shape of the impact damage region is obtained by the C-scan result. Thus, impact energy of 10 J is adopted to introduce impact damage in case C.

**Figure 13:**
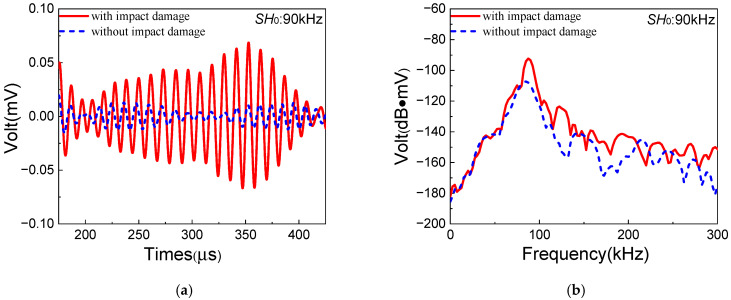
Resonant wave signals with the frequency 90 kHz generated from *S*_0_-*SH*_0_ mixing experiments in composite laminates with/without impact damage: (**a**) time domain; (**b**) frequency domain.

Similarly to case B, the signals of resonant waves and the corresponding ANPs are obtained to investigate the sensitivity of the *S*_0_-*SH*_0_ mixing method to impact damage in composite laminates. The signals of resonant waves with/without impact damage are shown in Figure 13. Compared with the case without impact damage, a more distinct diamond-shaped waveform of resonant waves could be discovered in the case with impact damage. Meanwhile, the ANPs without/with impact damage are denoted as $\beta_{i}$ and $\beta_{p}$, respectively. Impact damage in composite laminates is also cumulatively generated on the basis of inherent quadratic nonlinearity. In addition, the ANP focused on impact damage is denoted as $\beta_{p}^{\prime }=\beta_{p}-\beta_{i}$. The experimental values of $\beta_{i}$ and $\beta_{p}^{\prime }$ listed in Table 8 are 2.93 and 21.38, respectively, which means that ANP is significantly increased with impact damage. Therefore, it could be concluded that the one-way *S*_0_-*SH*_0_ mixing method has a remarkable effect on the detection of impact damage in composite laminates.

**Figure 14:**
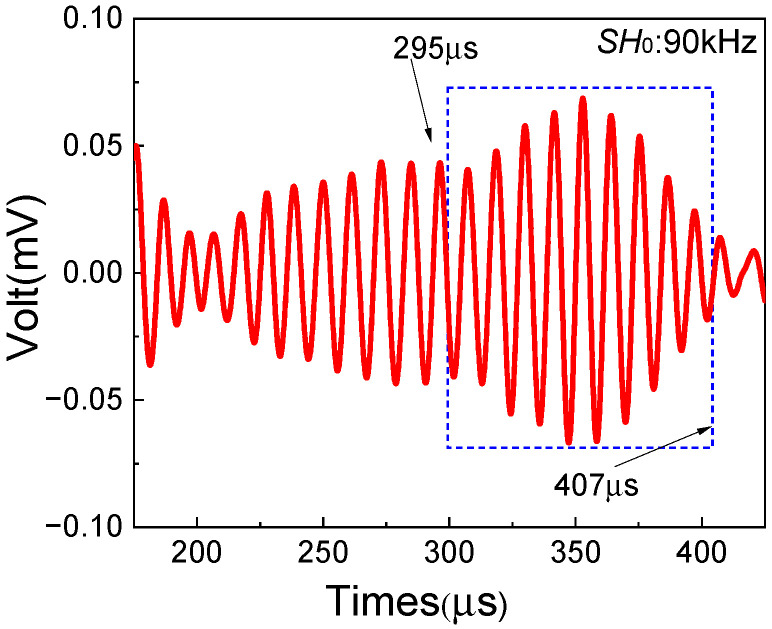
Resonant wave signal with the frequency 90 kHz from the impact damage region. The left and right ends of the blue dashed box indicate the start and end times of the resonant wave signal.

In addition, the damage localization method mentioned in Section 2 is employed to detect the impact damage region in composite laminates. Based on the theoretical arrival time of resonant waves and the distinct diamond-shaped waveform, the signals of resonant waves generated in the impact damage region are shown in Figure 14 (in the blue rectangular box). Then, the start and end time of resonant waves can be obtained, which are 295 μs and 407 μs, respectively. Based on the damage localization formula, the damage localization results could be calculated, as shown in Table 9.

**Figure 15:**
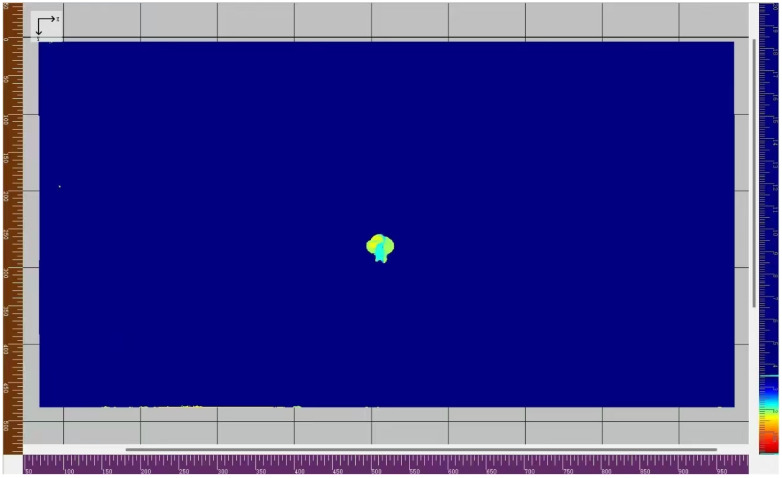
C-scan result of impact damage in T300 composite laminates.

Moreover, the wheel-mounted ultrasonic fast C-scan system is employed to verify the reliability of the damage localization method [58]. The system consists of a 128-element linear array wheel-mounted composite material probe, with an excitation frequency of 5 MHz ultrasonic waves. The handheld probe moves at a speed of 50 mm/s along the surface of composite laminates to acquire ultrasonic data for full coverage. The gate range is set to extract the reflected wave signal occurring between the initial pulse and the first full-thickness reflection pulse. Then, these data are processed by the C-scan system to generate C-scan images, which visually characterize the size, location, and depth of any damage. As a result, a two-dimensional cloud map is generated, as shown in Figure 15. The damage localization results from the C-scan method are also listed in Table 9.

Considering the damage localization results in Table 9, the positions of the damage center from the *S*_0_-*SH*_0_ mixing method and C-scan method are strongly consistent, being 513.15 mm and 512.31 mm, respectively. However, the size of the impact damage region from the *S*_0_-*SH*_0_ mixing method (118.10 mm) is obviously larger than that from the C-scan method (35.42 mm). It could be caused by the differences in fundamental principles between the *S*_0_-*SH*_0_ mixing method and the C-scan method. Due to impact damage, the transition region with material degradation may exist between a large-sized defect region and an undamaged region. Based on the linear reflected signals, only a large-sized defect region could be imaged by the C-scan method [59]. Nevertheless, both the transition region and large-sized defect region could lead to nonlinear ultrasonic signals based on the *S*_0_-*SH*_0_ mixing method. Thus, the larger size of the impact damage region could be obtained by the *S*_0_-*SH*_0_ mixing method, with high sensitivity to the transition region with material degradation. In conclusion, the one-way *S*_0_-*SH*_0_ mixing method could be a potential and effective technique for locating the impact damage region.

## 4. Conclusions

In this paper, the one-way *S*_0_-*SH*_0_ mixing method in composite laminates with inherent quadratic nonlinearity, delamination damage and impact damage, respectively, are investigated by a series of experiments. The damage localization method based on the one-way *S*_0_-*SH*_0_ mixing method is also experimentally verified. The following conclusions are drawn accordingly:

Firstly, in composite laminates with inherent quadratic nonlinearity, the resonant *SH*_0_ wave can be generated by a pair of *S*_0_-mode Lamb waves and *SH*_0_ waves when the resonance condition $\omega_{S_{0}}/\omega_{SH_{0}}=2\kappa /\left(\kappa +1\right)$ is satisfied. The resonant wave propagates in the opposite direction of fundamental waves. The frequency of the resonant wave is equal to the difference between two fundamental wave frequencies.

Secondly, because the vibration direction of the *SH*_0_ resonant wave is perpendicular to the direction of delamination type I, the one-way *S*_0_-*SH*_0_ mixing method is not sensitive to the delamination damage in composite laminates. However, the one-way *S*_0_-*A*_0_ mixing method could be more suitable for the detection of delamination damage in composite laminates than the one-way *S*_0_-*SH*_0_ mixing method.

Finally, the method to evaluate and locate the impact damage region is experimentally demonstrated based on the one-way *S*_0_-*SH*_0_ mixing method. As a contrast, the C-scan method is also employed to verify the feasibility of the damage localization method in this paper. Therefore, this study provides the experimental foundation for the one-way Lamb and SH mixing method to evaluate and locate the impact damage region in composite laminates.

## Figures and Tables

**Table 1:** Material parameters of T300 composite laminates.

Material	*ρ* (kg/m^3^)	*E*_1_ (GPa)	*E*_2_ (GPa)	*E*_3_ (GPa)	*ν*_12_	*ν*_1__3_	*ν*_23_	*G*_12_ (GPa)	*G*_13_ (GPa)	*G*_23_ (GPa)
T300	1760	139.92	10.05	10.05	0.31	0.31	0.48	5.70	3.40	3.40

**Table 2:** The representative frequency pairs of two fundamental waves in T300 composite laminates for experiments.

$f_{S_{0}}$ (kHz)	$f_{SH_{0}}$ (kHz)	$f_{R}$ (kHz)
220	140	80
250	160	90
330	210	120

**Table 3:** Theoretical and experimental group velocities of *SH*_0_ fundamental waves with different frequencies.

Frequency (kHz)	Group Velocity (m/s) (Experiment)	Group Velocity (m/s) (Theory)	Errors
140	1688.23	1799.62	6.19%
160	1720.54	1799.62	4.93%
210	1763.87	1799.62	1.98%

**Table 4:** Theoretical and experimental group velocities of *S*_0_-mode fundamental waves with different frequencies.

Frequency (kHz)	Group Velocity (m/s) (Experiment)	Group Velocity (m/s) (Theory)	Errors
220	6198.82	6267.48	1.09%
250	6098.64	6125.09	0.43%
330	5134.28	5062.67	1.41%

**Table 5:** The errors between experimental and theoretical frequencies of three resonant waves, corresponding to three frequency pairs of fundamental waves.

$f_{S_{0}}$ (kHz)	$f_{SH_{0}}$ (kHz)	$f_{R}$ (kHz) (Experiment)	$f_{R}$ (kHz) (Theory)	Errors
220	140	77.5	80	3.13%
250	160	90	90	0%
330	210	115	120	4.17%

**Table 6:** Damage localization results with different delay times. *P**_S_***—start position, *P**_E_***—end position.

∆*T* (μs)	*P_S_* (mm) (Theory)	*P_S_* (mm) (Experiment)	Errors	*P_E_* (mm) (Theory)	*P_E_* (mm) (Experiment)	Errors
120	155.2	170.6	9.92%	361	382.6	5.98%
200	345.7	334.3	3.30%	536.3	514.1	4.14%
260	496	527.5	6.35%	744.9	702.0	5.75%

**Table 7:** Comparison of ANPs obtained from *S*_0_-*SH*_0_ and *S*_0_-*A*_0_ mixing methods.

Method	$\beta_{d}$	$\beta_{i}$	$\beta_{d}^{\prime }$
*S*_0_-*SH*_0_	2.56	3.24	−0.68
*S*_0_-*A*_0_	4.39	2.79	2.6

**Table 8:** Comparison of ANPs obtained from *S*_0_-*SH*_0_ mixing experiments in composite laminates without/with impact damage.

$\beta_{i}$	$\beta_{p}^{\prime }$
2.93	21.38

**Table 9:** Damage localization results of *S*_0_-*SH*_0_ mixing method and C-scan method.

Position	*S*_0_-*SH*_0_ Mixing Method	C-Scan Method	Errors
*P_S_* (mm)	454.10	494.60	8.59%
*P_E_* (mm)	572.20	530.02	7.95%
*L* (mm)	118.10	35.42	233.43%
Damage center (mm)	513.15	512.31	0.16%

## Data Availability

Data will be made available on request.

## References

[1] Wang Q., Ma C., Yang D., Lu S., Liu X., Guo Y., Hu X. (2023). Quantitative monitoring of impact damage to composite structures using blade coated MXene sensors. Compos. Commun..

[2] Croxford A.J., Wilcox P.D., Drinkwater B.W., Nagy P.B. (2009). The use of non-collinear mixing for nonlinear ultrasonic detection of plasticity and fatigue. J. Acoust. Soc. Am..

[3] Pieczonka L., Ambrozinski L., Staszewski W.J., Barnoncel D., Pérès P. (2017). Damage detection in composite panels based on mode-converted Lamb waves sensed using 3D laser scanning vibrometer. Opt. Lasers Eng..

[4] Li W., Cho Y., Achenbach J.D. (2012). Detection of thermal fatigue in composites by second harmonic Lamb waves. Smart Mater. Struct..

[5] Li W., Wei Q., Huang J.K., Xiang L.S., Liu B. (2023). Nonlinear frequency mixing of counter-propagating collinear A0-S0 mode Lamb waves for delamination detection in CFRP composite plate. Nondestruct. Test. Eval..

[6] Raisutis R., Kazys R., Zukauskas E., Mazeika L., Vladisauskas A. (2010). Application of ultrasonic guided waves for non-destructive testing of defective CFRP rods with multiple delaminations. NDT&E Int..

[7] Soleimanpour R., Ng C.T. (2017). Locating delaminations in laminated composite beams using nonlinear guided waves. Eng. Struct..

[8] Šedek J., Šedková L., Vích O. (2025). Frequency-integral method for impact damage detection in carbon fibre reinforced thermoplastic composites by Lamb waves. Ultrasonics.

[9] Qu J., Xu C., Meng S. (2023). Experimental investigation on interlaminar and in-plane shear damage evolution of 2D C/SiC composites using acoustic emission and X-ray computed microtomography. Ceram. Int..

[10] Gao J., Fezzaa K., Chen W. (2022). Multiscale dynamic experiments on fiber-reinforced composites with damage assessment using high-speed synchrotron X-ray phase-contrast imaging. NDT&E Int..

[11] Chrysafi A.P., Athanasopoulos N., Siakavellas N.J. (2017). Damage detection on composite materials with active thermography and digital image processing. Int. J. Therm. Sci..

[12] Tuo H., Wu T., Lu Z., Ma X., Wang B. (2022). Study of impact damage on composite laminates induced by strip impactor using DIC and infrared thermography. Thin-Walled Struct..

[13] Lu H., Zheng T., Zhang L., Huang K., Liu X., Han X., Wang Y., Guo L. (2024). Damage identification of plain-woven composites at T > Tg using AE: Damage clustering and initiation detection. Compos. Sci. Technol..

[14] Saleem M., Khan B.S.H., Arumugam V. (2024). Damage and failure assessment of banana/ramie/epoxy composites using acoustic emission monitoring. Constr. Build. Mater..

[15] Li J., Lu Y., Guan R., Qu W. (2017). Guided waves for debonding identification in CFRP-reinforced concrete beams. Constr. Build. Mater..

[16] Ouvrier-Buffet F., Eiras J.N., Garnier V., Payan C., Ranaivomanana N., Durville B., Marquie C. (2021). Linear and nonlinear resonant ultrasonic techniques applied to assess delayed ettringite formation on concrete samples. Constr. Build. Mater..

[17] Luo L., Fan J., Ma J., Chen W., Song W., Bao S. (2024). A nonlinear ultrasonic wave mixing method for looseness detection of bolted joints. Ultrasonics.

[18] Quan S., Zhang Y., Lin P. (2023). Fatigue damage quantitative evaluation of carbon fiber composites at different stress ratios based on nonlinear ultrasonic. Results Phys..

[19] Sampath S., Liu H., Tham Z.W., Chen Y.F., Zhang L. (2024). Depth profiling of residual stress distribution in surface treated metallic structures using nonlinear ultrasonics. Ultrasonics.

[20] Srivastava A., Lanza di Scalea F. (2010). On the existence of longitudinal or flexural waves in rods at nonlinear higher harmonics. J. Sound Vib..

[21] Wang K., Liu M., Su Z., Guo S., Cui F. (2021). Mode-mismatching enhanced disbond detection using material nonlinearity in guided waves at low frequency. J. Sound Vib..

[22] Aseem A., Ng C.T. (2023). Collinear nonlinear guided wave mixing for debonding detection in reinforced concrete beam using longitudinal and torsional wave modes. Constr. Build. Mater..

[23] Gao X., Qu J. (2020). Necessary and sufficient conditions for resonant mixing of plane waves in elastic solids with quadratic nonlinearity. J. Acoust. Soc. Am..

[24] Weiss F.J., Kim J.-Y., Kurtis K.E., VanderLaan D., Tenorio C.N., Jacobs L.J. (2024). Experimental study on the nonlinear mixing of ultrasonic waves in concrete using an array technique. NDT&E Int..

[25] Li W., Jiang C., Xiao J., Xu C., Deng M. (2023). Assessment of Thermal Damage in Polymethyl Methacrylate Using Quasi-Static Components of Ultrasonic Waves. J. Nondestruct. Eval..

[26] Sun X., Liu H., Zhao Y., Qu J., Deng M., Hu N. (2020). The zero-frequency component of bulk waves in solids with randomly distributed micro-cracks. Ultrasonics.

[27] Shan S., Zhang C., Wu G., Song Y., Liu Z., Zhang Y., Cheng L. (2024). Amplitude-dependent second harmonic Lamb waves for discriminating delamination from background nonlinearities in composite plates. NDT&E Int..

[28] Rauter N., Lammering R., Kühnrich T. (2016). On the detection of fatigue damage in composites by use of second harmonic guided waves. Compos. Struct..

[29] Alnuaimi H., Amjad U., Park S., Russo P., Lopresto V., Kundu T. (2022). An improved nonlinear ultrasonic technique for detecting and monitoring impact induced damage in composite plates. Ultrasonics.

[30] Wang J., Lai Q., Xu C., Hu N., Deng M. (2023). High-frequency ultrasound-based thickness measurement of highly attenuating materials. Meas. Sci. Technol..

[31] Jiang C., Zhang C., Li W., Deng M., Ng C.-T. (2022). Assessment of damage in composites using static component generation of ultrasonic guided waves. Smart Mater. Struct..

[32] Jiao J., Sun J., Li G., Wu B., He C. (2015). Evaluation of the intergranular corrosion in austenitic stainless steel using collinear wave mixing method. NDT&E Int..

[33] Ju T., Achenbach J.D., Jacobs L.J., Qu J. (2019). Nondestructive evaluation of thermal aging of adhesive joints by using a nonlinear wave mixing technique. NDT&E Int..

[34] Sun M., Qu J. (2020). Analytical and numerical investigations of one-way mixing of Lamb waves in a thin plate. Ultrasonics.

[35] Lissenden C.J. (2021). Nonlinear ultrasonic guided waves—Principles for nondestructive evaluation. J. Appl. Phys..

[36] Li F., Zhao Y., Cao P., Hu N. (2018). Mixing of ultrasonic Lamb waves in thin plates with quadratic nonlinearity. Ultrasonics.

[37] Ding X., Xu C., Deng M., Zhao Y., Bi X., Hu N. (2021). Experimental investigation of the surface corrosion damage in plates based on nonlinear Lamb wave methods. NDT&E Int..

[38] Jiao J., Li L., Zhang H., Lv H., Wu B., He C. (2024). Ultrasonic immersion testing of residual stress in plates using collinear Lamb wave mixing technique. NDT&E Int..

[39] Zhang C., Li W., Deng M. (2024). Experimental observation of zero-group velocity combined harmonic generated by counter-directional Lamb wave mixing. Ultrasonics.

[40] Zhu H., Ng C.T., Kotousov A. (2022). Low-frequency Lamb wave mixing for fatigue damage evaluation using phase-reversal approach. Ultrasonics.

[41] Liu Y., Zhao Y., Deng M., Shui G., Hu N. (2022). One-way Lamb and SH mixing method in thin plates with quadratic nonlinearity: Numerical and experimental studies. Ultrasonics.

[42] Shan S., Hasanian M., Cho H., Lissenden C.J., Cheng L. (2019). New nonlinear ultrasonic method for material characterization: Codirectional shear horizontal guided wave mixing in plate. Ultrasonics.

[43] Li W., Xu Y., Hu N., Deng M. (2020). Impact damage detection in composites using a guided wave mixing technique. Meas. Sci. Technol..

[44] Pineda Allen J.C., Ng C.T. (2023). Damage detection in composite laminates using nonlinear guided wave mixing. Compos. Struct..

[45] Lan Z., Saito O., Okabe Y. (2024). Delamination detection in CFRP laminates using a chirp guided wave mixing technique. NDT&E Int..

[46] Aslam M., Lee J. (2024). Nonlinear guided wave mixing in weld joints for detection of material nonlinearity. Thin-Walled Struct..

[47] Su Z., Yang C., Pan N., Ye L., Zhou L.-M. (2007). Assessment of delamination in composite beams using shear horizontal (SH) wave mode. Compos. Sci. Technol..

[48] Liu X., Zhao Y., Deng M., Hu N. (2024). Numerical studies of one-way Lamb and SH mixing method in composite laminates with transverse-isotropic quadratic nonlinearity. J. Phys. Conf. Ser..

[49] Chen Z.M., Tang G.X., Zhao Y.X., Jacobs L.J., Qu J.M. (2014). Mixing of collinear plane wave pulses in elastic solids with quadratic nonlinearity. J. Acoust. Soc. Am..

[50] Ghayour M., Hojjati M., Ganesan R. (2020). Effect of tow gaps on impact strength of thin composite laminates made by Automated Fiber Placement: Experimental and semi-analytical approaches. Compos. Struct..

[51] Juarez P., Leckey C.A.C. (2018). Aerogel to simulate delamination and porosity defects in carbon-fiber reinforced polymer composites. AIP Conf. Proc..

[52] Yelve N.P., Mitra M., Mujumdar P.M. (2017). Detection of delamination in composite laminates using Lamb wave based nonlinear method. Compos. Struct..

[53] Petit S., Bouvet C., Bergerot A., Barrau J.-J. (2007). Impact and compression after impact experimental study of a composite laminate with a cork thermal shield. Compos. Sci. Technol..

[54] Dafydd I., Khodaei Z.S. (2020). Analysis of barely visible impact damage severity with ultrasonic guided Lamb waves. Struct. Health Monit..

[55] Chen M., Huan Q., Su Z., Li F. (2019). A tunable bidirectional SH wave transducer based on antiparallel thickness-shear (d15) piezoelectric strips. Ultrasonics.

[56] Zitouni I., Rhimini H., Chouaf A. (2024). A Combined Newton-Bisection Approach for Calculating the Dispersion Curves in Anisotropic Multilayered Waveguides. J. Vib. Eng. Technol..

[57] Wang L., Yuan F.G. (2007). Group velocity and characteristic wave curves of Lamb waves in composites: Modeling and experiments. Compos. Sci. Technol..

[58] Sutcliffe M.P.F., Monroy Aceves C., Stronge W.J., Choudhry R.S., Scott A.E. (2012). Moderate speed impact damage to 2D-braided glass–carbon composites. Compos. Struct..

[59] Yang L., Wu D., Liu H., Lu L., Yang Z., You S., Chen X. (2025). Air-coupled transducer tilted C-scan for delamination introduced during the manufacturing process in CFRP plates. Nondestruct. Test. Eval..

